# Management of Intraocular Pressure Elevation After CO_2_ Laser-Assisted Sclerectomy Surgery in Patients With Primary Open-Angle Glaucoma

**DOI:** 10.3389/fmed.2021.806734

**Published:** 2021-12-24

**Authors:** Min Chen, Yuxiang Gu, Yumei Yang, Qi Zhang, Xin Liu, Kaijun Wang

**Affiliations:** ^1^Eye Center of the 2nd Affiliated Hospital, School of Medicine, Zhejiang University, Hangzhou, China; ^2^Zhejiang Provincial Key Lab of Ophthalmology, Hangzhou, China; ^3^Shangyu People's Hospital of Shaoxing, Shaoxing, China

**Keywords:** CO_2_ laser, deep sclerectomy, Nd:YAG laser, intraocular pressure, primary open-angle glaucoma

## Abstract

**Purpose:** To report the safety and efficiency of carbon dioxide (CO_2_) laser-assisted sclerectomy surgery (CLASS) in Chinese patients with primary open-angle glaucoma (POAG) and the management of unexpected postoperative intraocular pressure (IOP) elevation.

**Methods:** This was a prospective case series study. A total of 23 eyes from 23 patients with POAG who underwent CLASS were involved and followed-up for 12 months. The primary outcomes included the changes in best corrected visual acuity (BCVA), IOP, and medications before and after CLASS. The secondary outcomes were success rate and postoperative laser interventions.

**Results:** The mean age of the patient was 42.6 ± 16.0 years. There was no significant change in BCVA and visual field at baseline and 12 months after CLASS. The number of medications was significantly reduced after CLASS. The IOP was also significantly decreased and remained well controlled during the follow-up period, except for a transient elevation at 1 month postoperatively, due to the occurrence of peripheral anterior synechiae (PAS). Generally, 17 patients (73.9%) were treated with neodymium-doped yttrium aluminum garnet (Nd:YAG) laser synechiolysis to remove iris obstruction in the filtration site and seven patients (30.4%) underwent Nd:YAG laser goniopuncture to deal with scleral reservoir reduction. Only one patient (4.3%) received surgical repositioning due to iris incarceration. The complete success rate and total success rate at 12 months were 69.6 and 95.7%, respectively.

**Conclusion:** CLASS was a safe and effective approach for Chinese patients with POAG. Peripheral anterior synechiae (PAS), iris incarceration, and scleral reservoir reduction were common causes of unexpected postoperative IOP elevation. Individualized Nd:YAG laser intervention helps to improve the long-term outcomes after CLASS.

## Introduction

Glaucoma is the leading cause of irreversible blindness worldwide. Elevated intraocular pressure (IOP) is the major factor leading to visual field loss and optic atrophy (1). At present, the treatment of glaucoma is focused on reducing IOP through medications, laser, or surgery (2).

Conventional trabeculectomy (Trab) remains to be the gold standard for filtering glaucoma surgery (3). However, there are various potential complications, including hypotony, shadow anterior chamber, infection, cataract development, malignant glaucoma, fistula failure, and suprachoroidal hemorrhage, which have direct influence on the success of the surgery (4).

Nonpenetrating deep sclerectomy (NPDS) is an alternative filtering surgery. Compared with Trab, NPDS has a higher safety, but is more technically demanding (5). It was designed to reduce the resistance of aqueous humor outflow by removing the outer wall of Schlemm's canal and the external part of the adjacent trabecular meshwork tissue, while avoiding penetrating the anterior chamber (6). The tissue must be dissected deep enough to achieve effective percolation, which is difficult to manipulate by manual procedure. Perforation of the trabeculo-Descemet membrane (TDM) is the most common intraoperative complication (7).

Carbon dioxide (CO_2_) laser-assisted sclerectomy surgery (CLASS) is an optimized approach of NPDS. CLASS uses a CO_2_ laser to ablate the scleral tissue instead of manual procedure, leaving a thin and intact TDM just sufficient for aqueous percolation without penetrating to the anterior chamber. During the process, the energy can be accurately controlled and the depth and size of the ablating area can be adjusted (8). It has been successfully used to treat primary open-angle glaucoma (POAG) during the recent years, both in the Caucasian and in Chinese patients (9–11). Compared with other filtering surgeries, CLASS requires a shorter learning curve and less technical challenge and also achieves higher safety (12).

Despite these unique advantages, CLASS also has some complications, including peripheral anterior synechiae (PAS), iris incarceration, shallow anterior chamber, choroidal detachment, etc. (13). It was reported that the incidence of PAS and iris incarceration was relatively high after CLASS, which resulted in a temporary increase of IOP (13). However, the difference between PAS and iris incarceration was vaguely described among previous studies. Insufficient penetration of the aqueous humor due to the decrease in the scleral reservoir is another reason leading to IOP elevation (14). So far, there is a lack of consensus on postoperative management of CLASS and laser intervention before and after CLASS remains controversial. Antiglaucoma medication was still the common treatment to deal with postoperative IOP elevation for most of Chinese patients. If we failed to identify the specific causes of IOP elevation and give proper interventions in time, it might lead to failure of the surgery.

In this study, we described a series of patients with POAG who underwent CLASS. For the first time, we analyzed possible causes of postoperative IOP elevation and proposed an ultrasound biomicroscopy (UBM)-guided individualized interventions according to different situations. We attempt to give some enlightenment to postoperative management of CLASS, in order to improve the long-term outcomes for Chinese patients with POAG.

## Materials and Methods

### Study Population

This was a prospective case series study, involving 23 eyes of 23 patients with POAG, who underwent CLASS and completed 1 year follow-up. All the subjects were recruited from our eye center between August 2020 and October 2021. The inclusion criteria were: patients aged ≥ 18 years, diagnosed with POAG, and with uncontrolled IOP under maximum hypotensive medications. The exclusion criteria were: patients with other systemic or ocular disorders, trauma or secondary glaucoma, or history of any ocular surgery or laser treatment. The study was conducted in accordance with the Declaration of Helsinki. Ethical approval was obtained from the Ethics Committee of the Second Affiliated Hospital of Zhejiang University. A written informed consent was obtained from all the subjects before all the procedures.

### Surgical Procedure and Postoperative Management

All the CLASS procedures were performed by a single surgeon (KJ Wang). In details, a fornix-based conjunctival flap was created to expose the sclera, then a one-third thickness limbus-based 5 mm × 5 mm scleral flap was made, and extended by 1 mm into the clear cornea. Mitomycin C (MMC) (0.4 mg/ml) was applied under the conjunctival and scleral flaps for 3 min and the area was washed with 20 ml balanced salt solution (BSS). A 4 mm × 2 mm scleral lake was created at the posterior scleral bed using a commercially available OT-135P2 CO_2_ laser system (IOPtiMate, IOPtima Ltd., Ramat Gan, Israel), with the depth of approximately 90% scleral thickness. MMC was applied again on the scleral lake for 1 min and washed out by BSS. Then, the CO_2_ laser beam was applied to ablate the outer wall of the Schlemm's canal and the trabecular meshwork, about 4 mm × 1.4 mm, until a continuous fluid percolation was observed. Finally, the two corners of the scleral flap and the conjunctival flap were sutured with 10/0 nylon sutures.

For all the patients, tobramycin and dexamethasone were prescribed postoperatively four times a day (QID) for 1 month and pilocarpine eyedrop was used QID for 3 months to prevent PAS. IOP was measured by the same experienced technician at each visit with Goldmann applanation tonometry. UBM and gonioscopy examination were performed at 1 month (M), 3, 6, 9, 12 M, and other situations of IOP elevation.

### Management of PAS

Peripheral anterior synechiae appeared as a physical contact at the filtrating area by UBM examination. A neodymium-doped yttrium aluminum garnet (Nd:YAG) laser synechiolysis was performed in clinic to remove iris obstruction and reopen the filtration site. After miosis with 2% pilocarpine (Furuida Corporation Ltd., Shandong, China) and instillation of topical alcaine (S.A. Alcon-Couvreur N.V., Belgium, UK), the superior angle was visualized under the Hwang-Latina 5.0 SLT Lens (Ocular Instruments, Bellevue, Washington, USA). Nd:YAG laser (Visulas YAG III Combi, Carl Zeiss Meditec AG, Jena, Germany, UK) spots were applied on the area of PAS with a power of 3–5 mJ, until the iris retreated from the trabeculodescemetic window (TDW). We do not puncture the TDW at the same time.

### Management of Iris Incarceration

Patients with iris incarceration can be identified by UBM examination as a prolapsed iris or have a pear-shaped pupil. Surgical repositioning was performed under the microscope. After topical anesthesia with alcaine, a 23-G needle was inserted from the clear corneal limbus into the superior anterior chamber angle to pull the incarcerated iris centrally, until the pupil returns to circular.

### Management of Scleral Reservoir Reduction

Once the postoperative IOP raised above the desired target IOP or the evidence of scleral reservoir reduction was observed by UBM examination, a Nd:YAG laser goniopuncture was performed to enhance the IOP-lowing effect. The energy was set at 3–5 mJ to create very small tears in the TDW and allows aqueous humor to flow from the anterior chamber into the sclera reservoir.

### Therapeutic Outcomes and Success Criteria

The primary outcomes included the changes in best corrected visual acuity (BCVA), IOP, and antiglaucoma medications before and after CLASS. Fixed combination medications were recorded as two types of agents. The secondary outcomes were success rate and postoperative laser interventions.

“Complete success” was defined as IOP values ranging between 5 and 18 mm Hg and IOP reduction of ≥ 20% from the baseline, without antiglaucoma medications or any interventions (including laser treatment, surgical repositioning, or needling). “Qualified success” referred to IOP values within the above criteria after postoperative interventions (including laser treatment, surgical repositioning, or needling) or under the application of antiglaucoma medications. “Failure” was defined as IOP < 5 mm Hg or > 18 mm Hg despite postoperative intervention or medications, or IOP reduction of < 20% from the baseline, or underwent additional glaucoma surgery within 1 year.

### Statistical Analysis

Statistical analyses were performed with the GraphPad Prism version 8.0 software (GraphPad Software Incorporation, San Diego, California, USA). The sample size was calculated based on a power calculation (power = 0.80; *p* = 0.05) using standard deviations (SDs) obtained in a previous study (11) and 17 eyes per group were considered well suited for the purpose of this study. Quantitative data were expressed as mean values including the SD. Descriptive statistics were performed to calculate the demographic characteristics of the study cohort. Normality was tested by means of D'Agostino and Pearson normality test. The Kruskal–Wallis test followed by the Dunn's *post-hoc* analysis was used to compare IOP values and medications before and after CLASS. The Wilcoxon matched-pairs signed rank test was applied to compare BCVA and mean defect (MD) at baseline and 12 M after CLASS. The Mann–Whitney *U* test was used to compare parameters between the two groups. The chi-squared test was used to compare incidence of complications between the two groups. A *p*-value of < 0.05 was considered to be statistically significant.

## Results

### Baseline Characteristics and Changes in Visual Acuity

A total of 23 eyes with POAG that underwent CLASS were recruited in this study including 14 males (60.9%) and 9 females (39.1%). The mean age was 42.6 ± 16.0 years (range 20–68 years). There was no significant difference between the BCVA at baseline [0.4 ± 0.6 logarithm of the minimum angle of resolution (logMAR)] and 12 M (0.3 ± 0.4 logMAR) postoperation (*p* = 0.125, Wilcoxon matched-pairs signed rank test). Besides, these patients had no significant progress in visual field at 1-year follow-up. The MD was 17.2 ± 7.0 dB at baseline and 18.5 ± 8.4 dB at 12 M (*p* = 0.210, Wilcoxon matched-pairs signed rank test), respectively.

### Changes in IOP and Medications

**Figure 1:**
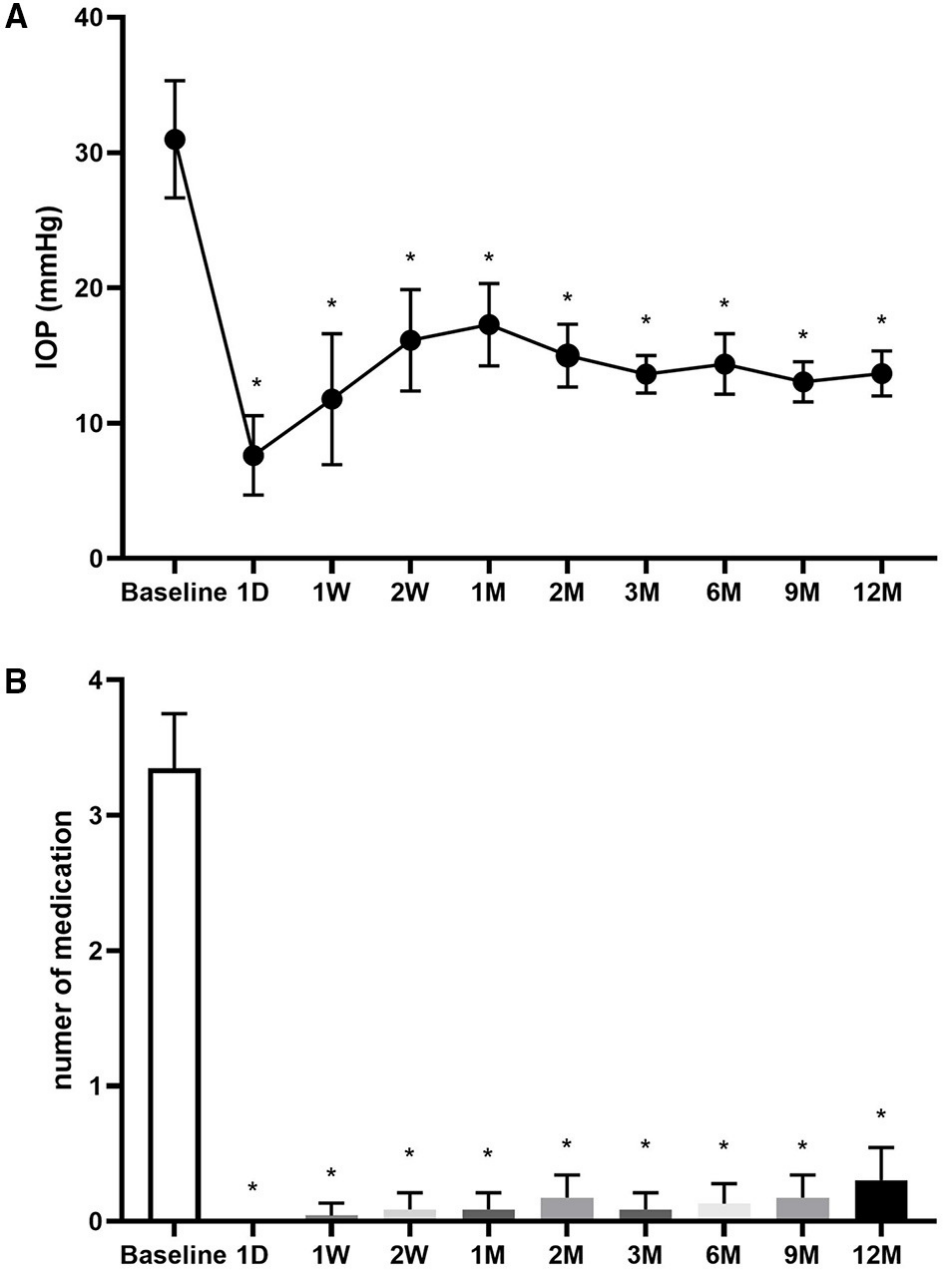
Changes in intraocular pressure (IOP) and number of medications. **(A)** Changes in IOP at baseline and 1 day (D), 1 week (W), 2 W, 1 month (M), 2, 3, 6 9, and 12 M after carbon dioxide (CO_2_) laser-assisted sclerectomy surgery (CLASS). **(B)** Changes in number of medications at baseline and 1 D, 1 W, 2 W, 1, 2, 3, 6, 9, and 12 M after CLASS. *Compared with baseline, *p* < 0.001, Kruskal–Wallis test followed by the Dunn's *post-hoc* analysis.

The baseline IOP was 31.0 ± 10.0 mm Hg, which significantly decreased to 11.8 ± 11.2 mm Hg at 1 week (W) after CLASS and gradually increased to 17.3 ± 7.0 mm Hg at 1 M, due to the occurrence of PAS. Through prompt intervention by Nd:YAG laser synechiolysis, the IOP decreased and remained well controlled during the subsequent follow-ups (Figure 1A).

The number of medications was 3.4 ± 0.9 before operation. After CLASS, most of the patients did not need to use antiglaucoma medications (Figure 1B). At 12 M postoperatively, the mean medication was only 0.3 ± 0.6 (*p* < 0.0001, compared with baseline, the Kruskal–Wallis test followed by Dunn's *post-hoc* analysis).

### Intraocular Pressure Elevation and Postoperative Interventions

**Figure 2:**
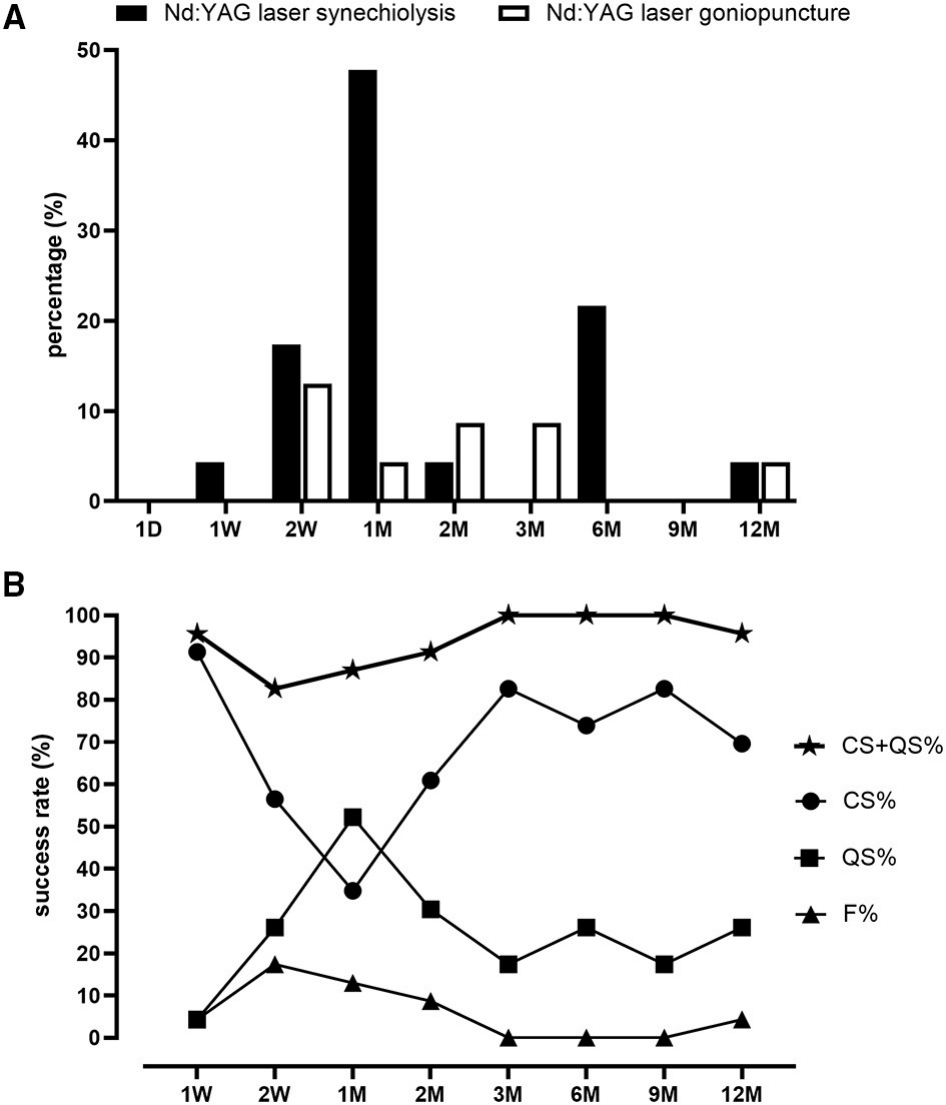
Proportions of postoperative laser interventions and success rate after CLASS. **(A)** Proportions of patients underwent neodymium-doped yttrium aluminum garnet (Nd:YAG) laser synechiolysis and Nd:YAG laser goniopuncture at different timepoints within 1 year follow-up. **(B)** Changes of complete success (CS) rate, qualified success (QS) rate, total success rate (QS + CS), and failure rate (F) at different timepoints within 1 year follow-up.

Postoperative interventions of the patients are given in Supplemental Table 1. PAS most often occurred at 1 M after CLASS (11 in 23 eyes, 47.8%, Figure 2A), which was the main cause of postoperative IOP elevation. Among the 23 patients, six patients developed PAS twice and two patients were treated with Nd:YAG laser goniopuncture twice. Data of the first treatment were included for analysis. Generally, 17 patients (73.9%) were treated with Nd:YAG laser synechiolysis due to PAS and seven patients (30.4%) underwent Nd:YAG laser goniopuncture to deal with scleral reservoir reduction during the 1-year follow-up. The IOP was significantly reduced after laser interventions immediately and the effect lasted for at least 3 months (Table 1).

**Table 1:** Comparisons of patients treated with Nd:YAG laser after CO_2_ laser-assisted sclerectomy surgery (CLASS).

	**Nd:YAG laser synechiolysis**	**Nd:YAG laser goniopuncture**	***P*-value^Δ^**
*N*	17	7	
Female/Male	6/11	3/4	>0.999
Age	43.2 ± 17.4	36.9 ± 12.6	0.426
Number of medications	3.5 ± 0.8	2.9 ± 1.2	0.228
Weeks after CLASS	6.1 ± 6.9	12.1 ± 16.2	0.299
Baseline IOP^a^	39.7 ± 11.8	26.4 ± 10.2	0.565
Pre IOP^b^	25.4 ± 11.2	20.8 ± 6.4	0.390
Post IOP^c^	12.9 ± 4.2^**^	11.8 ± 2.2^#^	0.458
1 M IOP^d^	13.0 ± 3.7^*^	9.3 ± 2.8^#^	0.108
3 M IOP^e^	13.1 ± 3.1^*^	10.5 ± 1.9^#^	0.164
Post IOP reduction%	43.6 ± 16.9	40.9 ± 13.8	0.939
1 M IOP reduction%	46.4 ± 21.1	47.8 ± 12.6	0.975
3 M IOP reduction%	42.3 ± 22.3	40.4 ± 7.2	0.832

*Nd:YAG, neodymium-doped yttrium aluminum garnet; IOP, intraocular pressure*.

a *Baseline IOP means IOP values before CLASS*.

b *Pre-IOP means IOP values before Nd:YAG laser treatment*.

c *Post-IOP means IOP values immediately after Nd:YAG laser treatment*.

d *1 M IOP means IOP values at 1 month after Nd:YAG laser treatment*.

e *3 M IOP means IOP values at 3 months after Nd:YAG laser treatment*.

** *Compared with pre-IOP, p < 0.0001,*

* *Compared with pre-IOP, p < 0.001,*

# *Compared with pre-IOP, p < 0.05, Kruskal–Wallis test followed by the Dunn's post-hoc analysis;*

Δ *Compared with the Mann–Whitney U test*.

**Figure 3:**
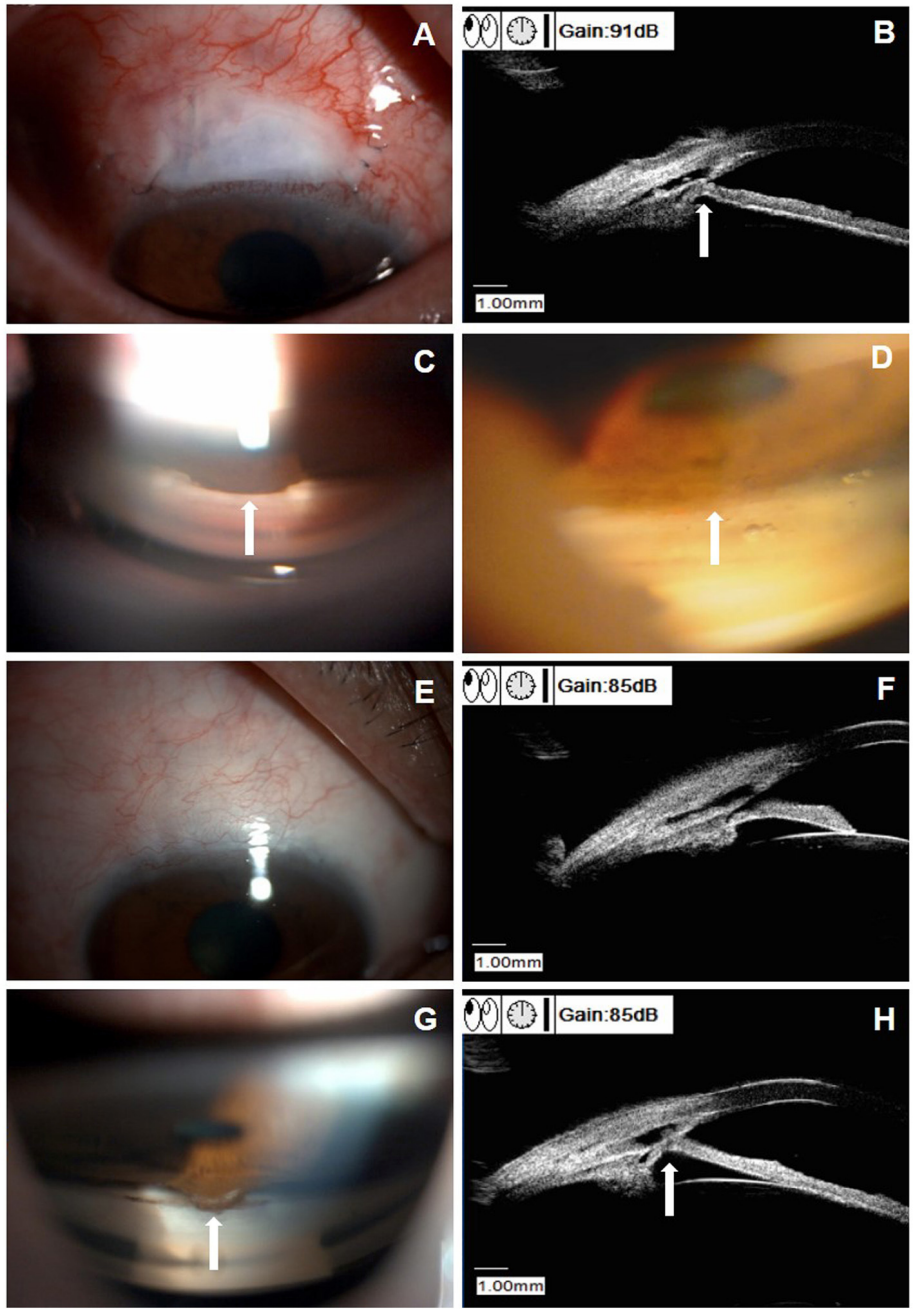
Clinical presentations of a patient (patient six in Supplemental Table 1) with peripheral anterior synechiae (PAS) after CLASS. **(A)** Slit-lamp examination (DC-4, Topcon Corporation, magnification 16X) of a 61-year-old male patient at 3 weeks after CLASS. **(B)** Ultrasound biomicroscopy (UBM) examination indicated PAS (white arrow). **(C)** Gonioscopy examination confirmed PAS around the treating area (white arrow). **(D)** After Nd:YAG laser synechiolysis treatment, the iris retreated from the trabeculodescemetic window (TDW) (white arrow). **(E)** Slit-lamp examination of the patient at 6 months after CLASS showed no obvious filtering bleb. **(F)** UBM examination at 6 months after CLASS showed no PAS. **(G)** Gonioscopy examination at 12 months after CLASS showed a second PAS (white arrow). **(H)** UBM examination confirmed a second PAS (white arrow) at 12 months after CLASS.

Figure 3 shows the images of a 61-year-old male patient (patient six in Supplemental Table 1), with a baseline IOP of 45.3 mm Hg under three antiglaucoma agents. He developed PAS at 3 weeks after CLASS and the IOP elevated to 20.5 mm Hg. After Nd:YAG laser synechiolysis treatment, the iris was retreated from the TDW and the IOP reduced to 10 mm Hg immediately. At 6 M postoperatively, there was no evidence of PAS from the UBM examination and the IOP was 15 mm Hg, without any medication. However, PAS occurred again at 12 M after CLASS and the IOP raised to 20.5 mm Hg. He underwent a second Nd:YAG laser synechiolysis treatment and the IOP dropped to 15 mm Hg immediately.

**Figure 4:**
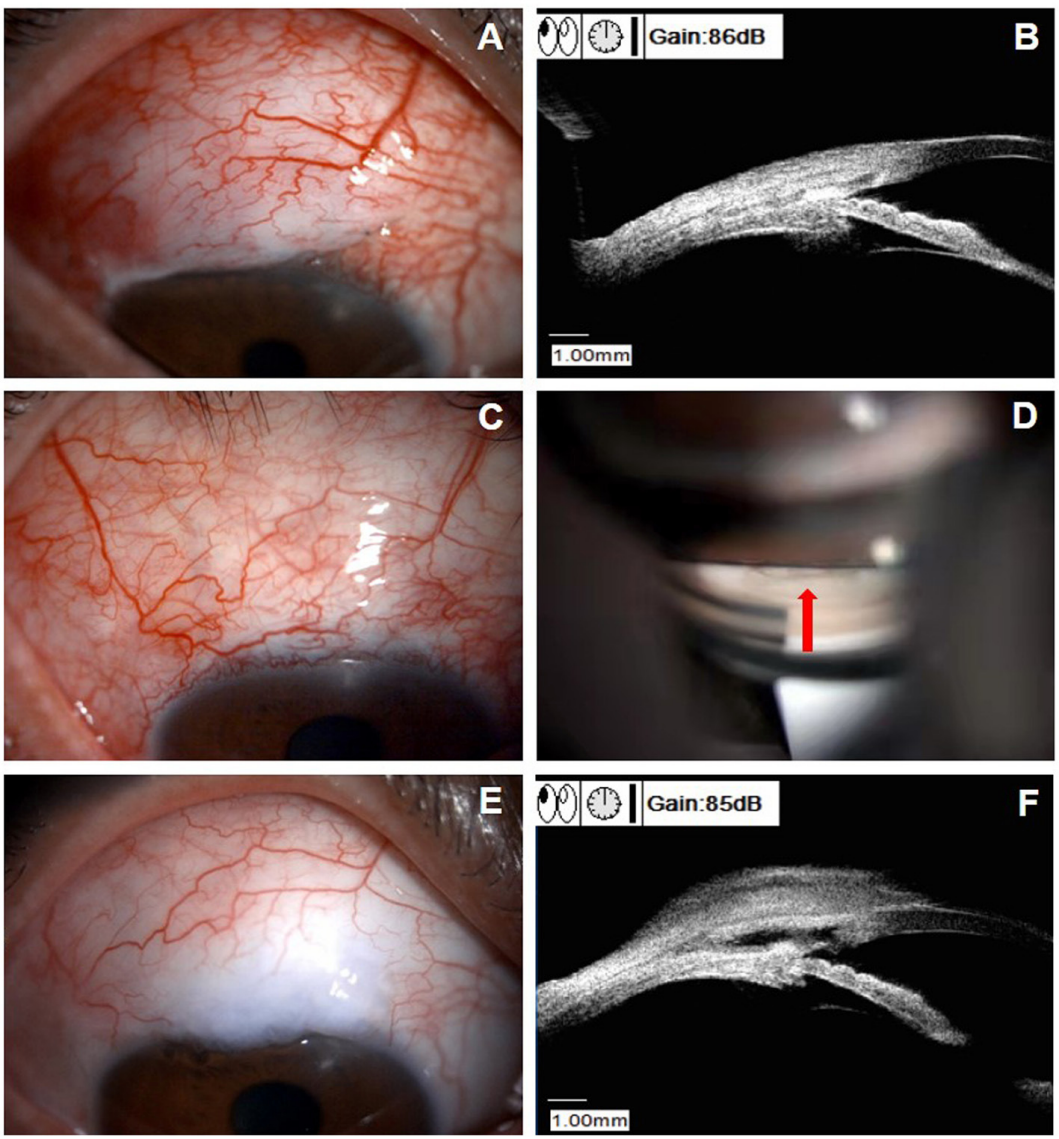
Clinical presentations of a patient (patient 23 in Supplemental Table 1) with scleral reservoir reduction. **(A)** Slit-lamp examination of a 46-year-old female patient at 1 month after CLASS, with a flat congested bleb at the filtering area. **(B)** UBM examination indicated a decrease in the scleral reservoir, although the IOP was 6.0 mm Hg. **(C)** Slit-lamp examination at 2 months after CLASS showed no obvious bleb with conjunctival congestion around the filtering area and the IOP raised to 25.7 mm Hg. **(D)** Gonioscopy examination indicated no PAS and Nd:YAG laser goniopuncture was performed at the TDW (red arrow) to enhance aqueous humor outflow. **(E)** Slit-lamp examination of the patient at 9 months after CLASS showed a flat and diffuse bleb. **(F)** UBM examination at 9 months after CLASS showed an enlarged scleral reservoir with a small hole in the TDW after Nd:YAG laser goniopuncture treatment.

Figure 4 displays the examinations of a 46-year-old female patient (patient 23 in Supplemental Table 1), who underwent an uneventful CLASS. The IOP decreased from 39.5 mm Hg at baseline to 6.0 mm Hg at 1 W postoperatively. At 1 M after CLASS, the IOP remained 6.0 mm Hg; however, UBM examination indicated reduction of the scleral reservoir. At 2 M after CLASS, the IOP raised to 25.7 mm Hg and gonioscopy examination indicated no PAS around the treating area. Then, a ND:YAG laser goniopuncture was performed and the IOP reduced to 13 mm Hg immediately. During the follow-up period, the patient developed a flat and diffuse bleb and the IOP was maintained 11–12 mm Hg without any medication.

### Therapeutic Outcomes

As shown in Figure 2B, the complete success (CS) rate was 91.3% at 1 W after CLASS and decreased to 56.5% at 2 W and only 34.8% at 1 M. Through prompt laser intervention, CS was gradually increased to 60.9% at 2 M, 82.6% at 3 M, 73.9% at 6 M, 82.6% at 9 M, and 69.6% at 12 M, respectively. Meanwhile, the qualified success (QS) rate increased from 4.3% at 1 W after CLASS to 52.2% at 1 M, which was gradually decreased during the subsequent follow-up. Total success rate (CS + QS) was over 80% at each timepoint (ranged from 82.6 to 100%). At 12 M after CLASS, the total success rate was 95.7%.

### Possible Factors Associated With PAS

We compared PAS incidence between young (< 45 years) and middle-aged to elderly patients (≥ 45 years). The results showed that the PAS incidence at 1 M was significantly higher in patients younger than 45 years (83.3%) than patients older than 45 years (18.2%, *p* = 0.003, chi-squared test, Table 2).

**Table 2:** Comparison of peripheral anterior synechiae (PAS) incidence after CLASS stratified by age and baseline IOP.

	**Age** **< 45 years**	**Age** **≥ 45 years**	***P*-value**	**Baseline IOP** **< 30 mm Hg**	**Baseline IOP** **≥ 30 mm Hg**	***P*-value**
N	12	11		12	11	
Age (years)	29.1 ± 6.4	57.3 ± 8.1	< 0.001^*^	44.8 ± 16.1	40.2 ± 16.4	0.66
Number of medications (mean ± SD)	3.3 ± 1.1	3.5 ± 0.8	0.76	3.3 ± 1.0	3.4 ± 0.9	0.89
Baseline IOP (mean ± SD)	30.1 ± 6.9	31.9 ± 12.9	1.00	23.6 ± 3.6	39.1 ± 8.3	< 0.0001^*^
PAS incidence at 1 month (%)	83.3%	18.2%	0.003^#^	33.3%	63.6%	0.22
PAS incidence within 1 month (%)	83.3%	45.5%	0.09	41.7%	81.8%	0.09
PAS incidence within 12 months (%)	83.3%	63.6%	0.37	66.7%	81.8%	0.64

* *Compared by the Mann–Whitney U test;*

# *Compared by the chi-squared (Fisher's exact) test*.

Then, PAS incidence was compared according to IOP. Our results showed that PAS incidence at the early stage (within 3 months) and within 12 months follow-up was higher, although not significantly, in patients with baseline IOP ≥ 30 mm Hg than patients with baseline IOP < 30 mm Hg (Table 2). Within 3 months after CLASS, 14 patients developed different degrees of PAS, while the other nine patients did not show any sign of PAS. Comparisons between the two groups showed no significant difference in mean age, baseline IOP, or IOP value at the first day after CLASS, but the degree of IOP reduction was significantly higher in patients with PAS (Table 3, *p* = 0.015, Mann–Whitney *U* test).

**Table 3:** Comparisons of patients with PAS within 3 months after CLASS.

	**With PAS**	**Without PAS**	***P*-value**
N	14	9	
Female/Male	9/5	5/4	
Age	42.2 ± 17.2	43.1 ± 15.1	0.988
Baseline IOP	33.9 ± 10.6	26.4 ± 7.5	0.074
1 D IOP	6.7 ± 4.4	9.0 ± 9.6	0.699
IOP reduction	27.2 ± 11.0	17.4 ± 8.5	0.015^*^
IOP reduction%	79.3 ± 12.0	67.7 ± 26.1	0.993

*IOP, intraocular pressure, D, day*.

* *Mann–Whitney U test, p < 0.05*.

### Surgical Complications

**Figure 5:**
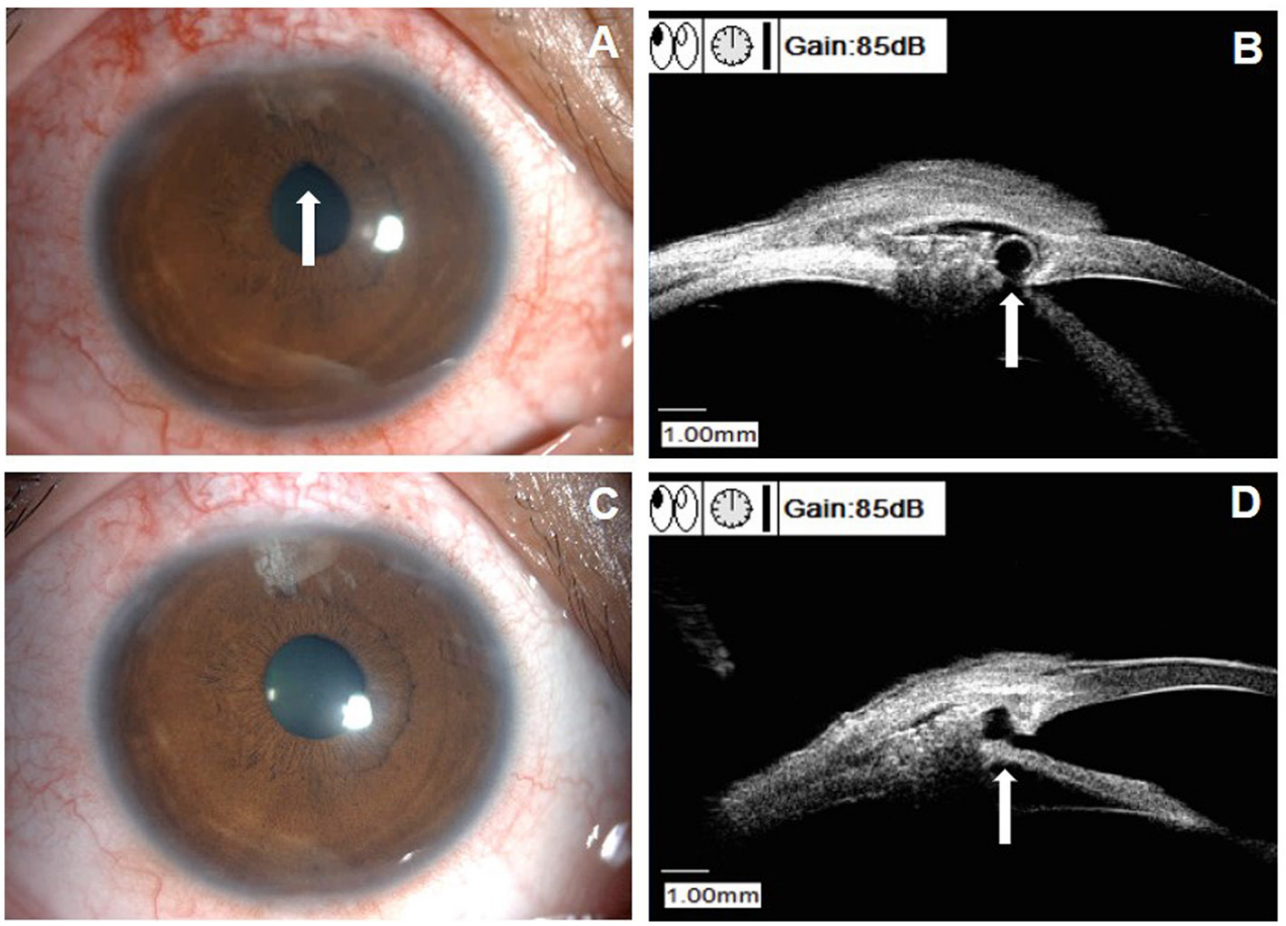
Clinical characteristics of a patient with iris incarceration (patient 10 in Supplemental Table 1). **(A)** Slit-lamp examination showed a pear-shaped pupil (white arrow). **(B)** UBM examination identified iris incarceration (white arrow). **(C)** Slit-lamp examination showed the pupil shape returned to circular after surgical repositioning. **(D)** UBM examination showed the iris root returned to normal site after surgical repositioning (white arrow).

Uneventful CLASS was performed in all the patients, expect for one patient who had intraoperative microperforation (4.3%). The patient developed iris incarceration and received surgical repositioning at 2 M after CLASS (Figure 5). During follow-ups, subconjunctival injection of 5-fluorouracil (5-FU) was performed in one patient (4.3%) and one patient (4.3%) developed shallow anterior chamber. No other complications were observed during the follow-up period.

## Discussion

Conventional trabeculectomy has been acknowledged to be the gold standard for glaucoma surgery (3). Considering the limited success rate and potential complications of Trab, efforts have been made to develop new surgical approaches. In the recent years, laser-assisted techniques have gained increasing attention and CO_2_ laser is most commonly used in glaucoma surgery (15). CO_2_ laser irradiation can achieve effective photoablation on dry tissue, but has a high absorption affinity for water such as aqueous humor (16). Due to its unique advantage, CO_2_ laser was first applied by Assia et al. as a simplified NPDS in 2007, named as CLASS (16).

Clinical studies have demonstrated its efficacy and safety in patients with POAG (12). Table 4 summarizes long-term outcomes of CLASS among published studies. The CS rate ranged from 45.5 to 67.9% at 12 M and ranged from 34.1 to 73.0% at 24 M. Meanwhile, the QS rate ranged from 69.2 to 93.1% at 12 M and ranged from 76.9 to 96.0% at 24 M. Compared between different populations, the mean CS at 12 M after CLASS was higher in Chinese patients (64.6%) than in Caucasian patients (44.9%). Zhang et al. conducted a study in a group of Chinese patients with POAG and reported a CS of 58.6% and QS of 93.1% at 12 M after CLASS (11). In this study, the CS was 69.8% at 1-year follow-up, which was better than their results. As shown in Supplemental Table 2, the definition of QS in this study was different from previous studies. Therefore, the value of (CS + QS) in this study (95.7%) was consistent with QS value in published literatures, which was also higher than previous studies (Table 4). In case of unexpected IOP elevation after CLASS, we preferred to reduce IOP by timely interventions (including laser treatment, surgical repositioning, or needling) according to different causes, rather than by antiglaucoma medications, which might contribute to the better long-term outcomes in this study.

**Table 4:** Comparison of long-term outcomes of CLASS among published studies.

**No**.	**First author**	**Year**	**Population**	**Surgery**	**N** **(eye)**	**PAS** **incidence (%)**	**Iris** **incarceration (%)**	**Complete success rate (%)**				**Qualified success rate (%)**			
								**6 M**	**12 M**	**24 M**	**36 M**	**6 M**	**12 M**	**24 M**	**36 M**
1	Geffen et al.	2010	Mixed	CLASS	37	0.0%	0.0%	76.7%	60.0%	—	—	83.3%	86.6%	—	—
2	Greifner et al.	2014	Caucasian	CLASS	27	0.0%	48.0%	—	—	73.0%	—	—	—	96.0%	—
				NPDS	31	0.0%	0.0%	—	—	71.0%	—	—	—	89.0%	—
3	Skaat et al.	2014	Caucasian	CLASS	15	0.0%	6.7%	—	45.5%	—	—	—	90.9%	—	—
4	Geffen et al.	2016	Mixed	CLASS	97	5.6%	8.3%	—	60.2%	57.9%	47.8%	—	79.6%	91.2%	84.8%
5	Yick et al.	2016	Chinese	CLASS	23	0.0%	0.0%	—	—	—	—	81.8%	—	—	—
6	Cutolo et al.	2017	Caucasian	CLASS	21	9.5%	14.3%	—	—	—	—	—	—	—	—
7	Yu et al.	2018	Chinese	CLASS + Phaco	17	0.0%	0.0%	—	65.0%	—	—	—	88.0%	—	—
8	Jankowska-Szmul et al.	2018	Caucasian	CLASS	66	0.0%	4.5%	—	35.0%	—	—	—	74.0%	—	—
				Trab	65	0.0%	3.1%	—	60.0%	—	—	—	75.0%	—	—
9	Villavicencio et al.	2018	Caucasian	CLASS + Phaco	33	0.0%	33.3%	—	—	—	—	—	97.2%	—	—
				Trab + Phaco	37	0.0%	0.0%	—	—	—	—	—	86.4%	—	—
10	Jankowska-Szmul et al.	2018	Caucasian	CLASS	66	3.0%	3.0%	—	35.0%	—	—	—	74.0%	—	—
11	Zhang et al.	2020	Chinese	Modified CLASS	25	6.9%	0.0%	—	62.1%	48.3%	—	—	89.7%	89.7%	—
12	Sohajda et al.	2020	Caucasian	CLASS	22	0.0%	0.0%	72.7%	64.0%	—	—	77.0%	72.7%	—	—
13	Yan et al.	2020	Chinese	CLASS	28	10.7%	0.0%	71.4%	67.9%	64.3%	—	92.9%	85.7%	85.7%	—
14	Zhang et al.	2021	Chinese	CLASS	30	30.0%	6.7%	82.8%	58.6%	51.7%	—	100.0%	93.1%	86.2%	—
				Trab	47	0.0%	0.0%	82.6%	60.0%	47.7%	—	95.7%	93.3%	84.1%	—
15	Ho et al.	2021	Asian	CLASS	13	0.0%	0.0%	41.5%	41.5%	34.1%	24.4%	48.8%	69.2%	76.9%	53.8%
				CLASS + Phaco	28	0.0%	0.0%	—	—	—	—	—	46.4%	53.6%	50.0%
16	Current study	2021	Chinese	CLASS	23	73.9%	4.3%	—	69.8%	—	—	—	95.7%	—	—

*PAS, peripheral anterior synechiae; Phaco, phacoemulsification; Trab, trabeculectomy; NPDS, nonpenetrating deep sclerectomy; IOP, intraocular pressure; M, months*.

Compared with Trab, CLASS had fewer complications, quicker visual recovery, and similar IOP lowering efficacy (11). As an optimized NPDS, CLASS was found to be a relatively easy procedure (13). Theoretically, CLASS offers the advantage of enhanced safety and accuracy under controllable laser ablation. However, the main drawback of laser-assisted surgery was potential collateral damage and tissue coagulation induced by the scattered energy (15). The most advanced system (OT-135P2, IOPtiMate) with a higher laser power and less beam dwell time was applied in this study to achieve effective ablation during the CLASS procedure and minimize residual momentary heating and tissue coagulation. Despite such improvement, postoperative complications such as PAS seemed to be inevitable after CLASS. As shown in Table 4, the incidence of PAS after CLASS ranged from 0.0 to 30.7% among previous studies. Compared with Caucasian patients, relatively higher rates of PAS were found in Chinese patients. Jankowska-Szmul et al. (9) reported only 3.0% PAS incidence and Cutolo et al. (17) reported 9.5% PAS incidence, while Zhang et al. showed a 30.0% PAS incidence after CLASS (11). In this study, the incidence of PAS was significantly higher than the abovementioned studies. Totally, 17 patients (73.9%) experienced different degrees of PAS during the follow-up period and almost half of the patient (47.8%) occurred PAS at 1 M postoperatively, which was the main cause of IOP elevation at the early stage after CLASS. We also found that the variation of PAS incidence was consistent with the fluctuation of CS and QS, which indicated that 1 M after CLASS was a critical timepoint for postoperative management.

The following factors might be associated with PAS after CLASS: (1) The TDM was a narrow and thin membrane near the iris root, which increased the risk of PAS (11). Recently, a modified CLASS was reported by Zhang et al., who created the TDW forward to the Schlemm's canal and relatively far from the iris root, in order to reduce the PAS incidence (18); (2) The intraoperative outflow and postoperative overfiltering promoted occurrence of PAS (11). Our results indicated a higher baseline IOP and a greater IOP reduction at the first day that might be potential risk factors of PAS. Therefore, we suggested that the IOP of the patient should be controlled lower than 30 mm Hg before operation to avoid rapid outflow of the aqueous humor during the surgery. Besides, the scleral flap should be sutured with appropriate tightness to prevent overfiltering at the early stage after CLASS; (3) Collateral thermal damage around the ablated area might lead to local inflammation and synechiae formation (11). Topical anti-inflammatory treatment was necessary during the early postoperative period; (4) A crowded anterior segment in Chinese patients with POAG (19), especially for those aged > 50 years (20), might be another reason for high PAS incidence. In this study, young patients (< 45 years) seemed to have a higher PAS incidence at the early stage after CLASS. Limited sample size and higher baseline IOP might be possible reasons for this inconsistency, which needed to be further confirmed in future studies.

Whether prophylactic laser treatment was needed before CLASS, it remains controversial so far. Zhang et al. conducted preventive laser peripheral iridotomy (LPI) and argon laser peripheral iridoplasty (ALPI) in 29 patients with POAG before a modified CLASS and reported only 6.9% of PAS incidence (18). Zhang et al. also performed LPI + ALPI in seven eyes with narrow angle before CLASS and six of them did not develop PAS postoperatively (11). In this study, none of the patient received prophylactic laser treatment before CLASS, which might be one of the reasons for the higher PAS incidence than previous studies. Based on our experience, PAS did not occur in all the patients after CLASS. Even if happened, it could be easily resolved by Nd:YAG laser intervention as long as it could be identified in time. Therefore, it is not necessary to add two invasive treatments to all the patients in order to prevent a potential complication. Also, it was difficult to ensure that the position of preoperative laser treatment was consistent with the laser ablation area. Besides, we found that PAS may occur at any time after CLASS and some patients experienced PAS more than once during the follow-up period. Regular follow-ups and individualized postoperative management were more important than prevention for these patients to achieve satisfactory long-term outcomes.

Iris incarceration was another cause of IOP elevation after CLASS. As shown in Table 5, the rate of iris incarceration ranged from 3.0 to 48.0% and only 4.3% (1/23) incidence of iris incarceration was found in this study. There was no clear definition of iris incarceration in published literatures. Sometimes, the incidence of PAS and iris incarceration were reported together. In this study, only one patient (patient 10 in Supplemental Table 1) developed iris incarceration after CLASS. She experienced PAS at 2 W and 1 M postoperatively and underwent Nd:YAG laser synechiolysis treatment twice. However, the IOP rose again with a pear-shaped pupil. Iris incarceration was identified by UBM examination and surgical repositioning was performed (Figure 5). We speculated that this patient developed microperforation during the surgery and previous laser intervention might cause small tears in the TDW, which aggravated iris incarceration. Therefore, carefully ablation and energy adjustment were critical to avoid perforation during the surgery. Also, laser spots should be applied on the attached iris when dealing with PAS without excessive puncture on the TDW. Besides, compression, massage of the eyeball, or severe cough should be avoid after CLASS, which might cause rupture of the thin TDW and the internal Schlemm's canal wall (11).

Reduction of the scleral reservoir was the third cause of postoperative IOP elevation after CLASS. Compared with Caucasian populations, fibrotic responses were more common in Chinese patients after glaucoma surgery (21, 22). Excessive coagulation around the ablating area might cause synechiae formation (23). For these reasons, either the “window” or the “lake” could scar down over time, which could be easily identified by UBM. Nd:YAG laser goniopuncture was an adjunctive procedure to achieve further reduction of IOP, by turning a nonpenetrative surgery into a micropenetrating surgery (24). It was reported that Nd:YAG laser goniopuncture could reduce the IOP by 42% after NPDS (25) and over 50% patients achieved IOP < 15 mm Hg without any hypotonic medication for at least 2 years (24). About 41 to 63% proportion of Nd:YAG laser goniopuncture treatment was reported in published studies (26, 27). In this study, 30.4% patients underwent Nd:YAG laser goniopuncture at 12.1 ± 16.2 weeks after CLASS and the IOP reduced by 40.4% at 3 months after the laser treatment. Despite the beneficial effect, Nd:YAG laser goniopuncture was associated with some potential complications. The most common one was iris incarceration, which occurred in 17.6% eyes underwent Nd:YAG laser goniopuncture (24). High IOP before the treatment and laser intervention within 3 months after NPDS were two predicting factors of iris incarceration (24). In this study, we did not observe any remarkable iris incarceration after Nd:YAG laser goniopuncture. Although both the laser interventions in this study were carried out with Nd:YAG laser shot, the sites were totally different. We do not recommend performing Nd:YAG laser synechiolysis and Nd:YAG laser goniopuncture at the same time when dealing with PAS because it may increase the risk of iris incarceration.

This study had several limitations. First, the sample size was relatively small and the follow-up period was limited. It is necessary to conduct a randomized prospective study with larger sample size and longer follow-up period to further evaluate the long-term safety and efficacy of CLASS. Second, control group was not included in this study, which made the results less impactful. Moreover, there was a lack of morphological classification of the bleb and quantitative comparison of the scleral reservoir, which could provide useful information to evaluate the mechanisms after CLASS. Besides, degree of PAS was not evaluated, which might be helpful to investigate the possible reasons behind the high incidence of PAS. Despite these limitations, we distinguished PAS from iris incarceration through UBM examination and proposed a UBM-guided individualized intervention. The effect and duration of Nd:YAG laser treatment were analyzed and the risk factors associated with PAS were also evaluated in this study. These have never been addressed in detail among previous studies. We hope that our preliminary results can fill up the study gap in this field and also provide a valuable reference for clinical treatment.

In conclusion, CLASS was a safe and effective approach for Chinese patients with POAG. PAS, iris incarceration, and reduction of scleral reservoir were common causes of unexpected postoperative IOP elevation. A UBM-guided individualized Nd:YAG laser intervention can not only resolve PAS at the early stage after CLASS, but also achieve further IOP reduction whenever necessary, which helps to improve the long-term outcomes after CLASS.

## Data Availability Statement

The original contributions presented in the study are included in the article/Supplementary Material, further inquiries can be directed to the corresponding author/s.

## Ethics Statement

The studies involving human participants were reviewed and approved by Ethics Committee of the Second Affiliated Hospital of Zhejiang University. The patients/participants provided their written informed consent to participate in this study. Written informed consent was obtained from the individual(s) for the publication of any potentially identifiable images or data included in this article.

## Author Contributions

MC and YG contributed to the design and concept of study, data collection and analysis, and drafting and revision of manuscript. KW contributed to the design and concept of study and performing operation. YY, XL, and QZ contributed to the data collection and analysis and interpreting results. All the authors listed have made a substantial contribution to the work and approved it for publication.

## Funding

This study was supported by the Zhejiang Provincial Public Welfare Technology research project (No. LGF20H120003) and National Natural Science Foundation of China (No. 82171045).

## Conflict of Interest

The authors declare that the research was conducted in the absence of any commercial or financial relationships that could be construed as a potential conflict of interest.

## Publisher's Note

All claims expressed in this article are solely those of the authors and do not necessarily represent those of their affiliated organizations, or those of the publisher, the editors and the reviewers. Any product that may be evaluated in this article, or claim that may be made by its manufacturer, is not guaranteed or endorsed by the publisher.
