# Supplementary material for: Management of Intraocular Pressure Elevation After CO2 Laser-Assisted Sclerectomy Surgery in Patients With Primary Open-Angle Glaucoma
Source: Front Med (Lausanne). 2021 Dec 24;8:806734. doi: 10.3389/fmed.2021.806734 (PMC8740123; doi:10.3389/fmed.2021.806734)
Supplement: Supplementary file 1 [file Data_Sheet_1.docx]

Supplementary Material

Supplemental Table 1. Summary of postoperative interventions of the patients after CO_2_ laser assisted sclerectomy surgery.


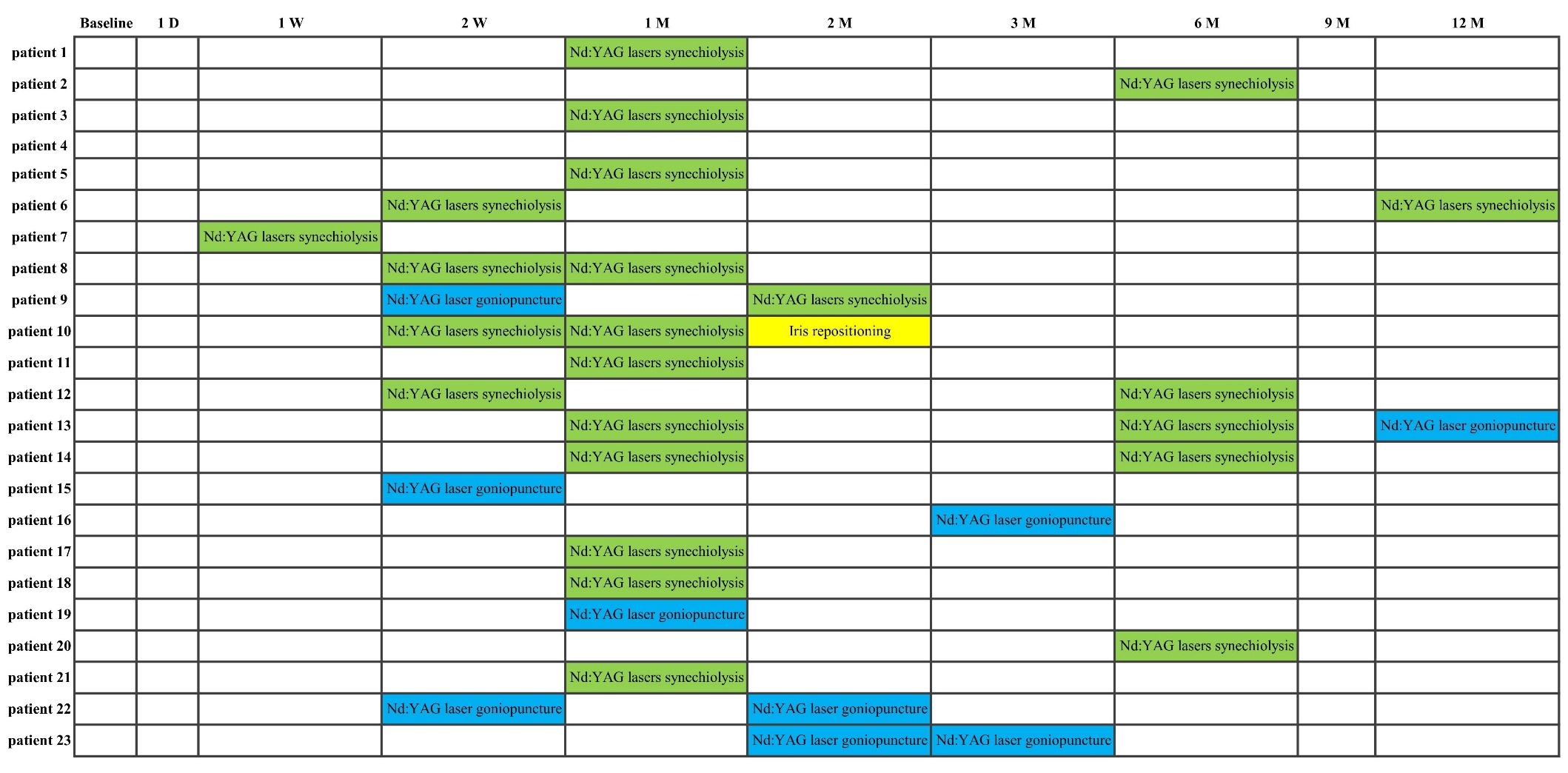


|  |  |  |  |  |  |  |  |  |  |  |
| --- | --- | --- | --- | --- | --- | --- | --- | --- | --- | --- |


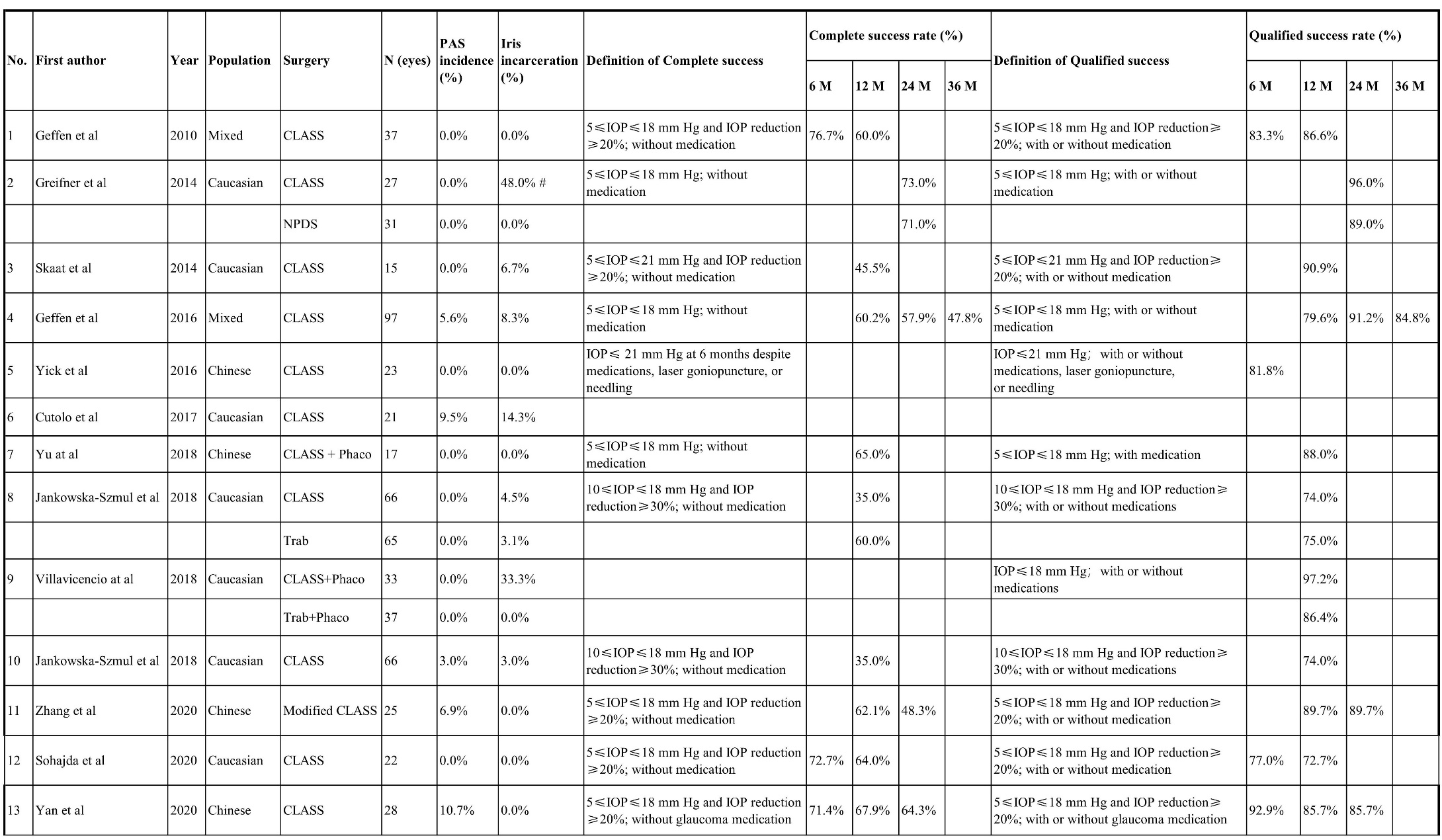
Supplemental Table 2. Comparison of long-term outcomes of CO_2_ laser assisted sclerectomy surgery among published studies.


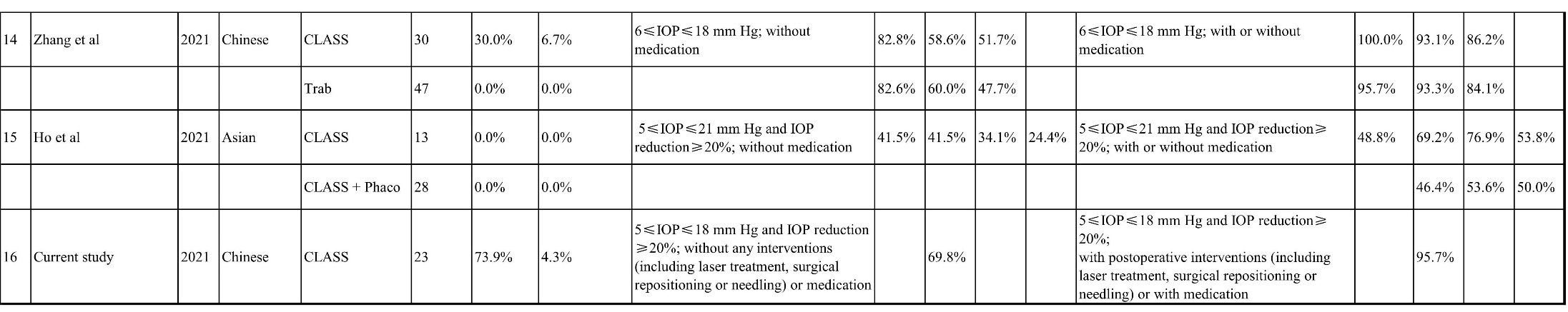


# 25.9% spontaneously, 22.1% after Nd:YAG laser goniopuncture; PAS: Peripheral anterior synechia; CLASS: CO_2_ laser assisted sclerectomy surgery; Phaco: Phacoemulsification; Trab: trabeculectomy; NPDS: non-penetrating deep sclerectomy; IOP: intraocular pressure. M: months
